# The Brain–Intestinal Mucosa–Appendix– Microbiome–Brain Loop

**DOI:** 10.3390/diseases6020023

**Published:** 2018-04-01

**Authors:** Luis Vitetta, Gemma Vitetta, Sean Hall

**Affiliations:** 1 Sydney Medical School, The University of Sydney, Sydney 2006, Australia; 2 Medlab Clinical Ltd., Sydney 2015, Australia; gemmavitetta@gmail.com (G.V.); sean_hall@medlab.co (S.H.)

**Keywords:** brain-intestinal-brain axis, intestinal epithelia, macrophages, vagus nerve, microbiome, dysbiosis, vermiform appendix, probiotics

## Abstract

The brain and the gut are connected from early fetal life. The mother’s exposure to microbial molecules is thought to exert in utero developmental effects on the fetus. These effects could importantly underpin the groundwork for subsequent pathophysiological mechanisms for achieving immunological tolerance and metabolic equilibrium post birth, events that continue through to 3–4 years of age. Furthermore, it is understood that the microbiome promotes cues that instruct the neonate’s mucosal tissues and skin in the language of molecular and cellular biology. Post birth mucosal lymphoid tissue formation and maturation (most probably including the vermiform appendix) is microbiota-encouraged co-establishing the intestinal microbiome with a developing immune system. Intestinal mucosal tissue maturation loops the brain-gut-brain and is postulated to influence mood dispositions via shifts in the intestinal microbiome phyla. A plausible appreciation is that dysregulated pro-inflammatory signals from intestinal resident macrophages could breach the loop by providing adverse mood signals via vagus nerve afferents to the brain. In this commentary, we further suggest that the intestinal resident macrophages act as an upstream traffic controller of translocated microbes and metabolites in order to maintain local neuro-endocrine-immunological equilibrium. When macrophages are overwhelmed through intestinal microbiome and intestinal epithelial cell dysbiosis, pro-inflammatory signals are sustained, which may then lead to mood disorders. The administration of probiotics as an adjunctive medicine co-administered with antidepressant medications in improving depressed mood may have biological and clinical standing.

## 1. Commentary

### 1.1. Macrophages and Intestinal Tissue

Both the intestines and the brain develop from the same cluster of embryonic tissue that can be traced back to the primitive streak in early vertebrate fetal growth. As such, this grooved structure that forms on day 15 of human development, along the caudal midline of the bilaminar embryonic disc is the first visible sign of gastrulation that will give rise to the ectoderm, mesoderm and endoderm [1]. When the tissue divides, a portion develops into the Central Nervous System (CNS) (i.e., brain and spinal cord) and the other into the Enteric Nervous System (ENS). Immune molecules are intimately related to the development of the CNS as they are for the intestinal tract. The intestinal tract is a complex multi-dimensional structure that is derived from a simple tubular structure [2]. Such is the complexity that the established intestinal epithelia are embraced by the crypt-villus elements that contain absorptive enterocytes and secretory tuft, goblet, Paneth, entero-endocrine cell types; while also accommodating resident intestinal stem cells and rapidly dividing progenitor cells. Adjacent to the intestinal epithelium is an additional complex structure comprising the lamina propria, submucosa, and muscular layers. 

Immune molecules have been reported to participate in integral functions in the CNS throughout various stages of neural development, including affecting neurogenesis, neuronal migration, axon guidance, synapse formation, activity-dependent refinement of circuits, and synaptic plasticity [3]. For example, the innate-like, evolutionarily conserved MR1-restricted mucosa-associated invariant T cells (MAIT) from humans represent the most abundant T-cell subset that quickly respond to a wide variety of bacteria [4]. MAIT cells in humans are prevalent and are distributed throughout the blood and mucosal sites [5]. Studies of MAIT cells in human fetal development are important for the consideration of the fundamental immune-biological characteristics expressed, as well as for the role these cells play in the fetus and the newborn. In humans T cells are enriched in mucosal tissues [6].

In the intestines there is recognized a tripartite co-operation in order to maintain intestinal steady state homeostasis. This occurs between the intestinal epithelium barrier, the intestinal microbiome and gut mucosal immune cells such as macrophages [2,7,8]. 

Macrophages (mononuclear phagocytes) are distributed throughout the body in tissue sites that include for example the intestines, the lung, the liver, and the brain [9]. Macrophages are established from prenatal signals and from circulating monocytes post birth [9]. The appreciation that macrophages have multiple actions in order to maintain the overall immunological efficiency in the gut is an idea that reflects macrophage functional diversity in specific tissues, where the tissue site provides instructive signals for local macrophage differentiation [10]. 

**Figure 1:**
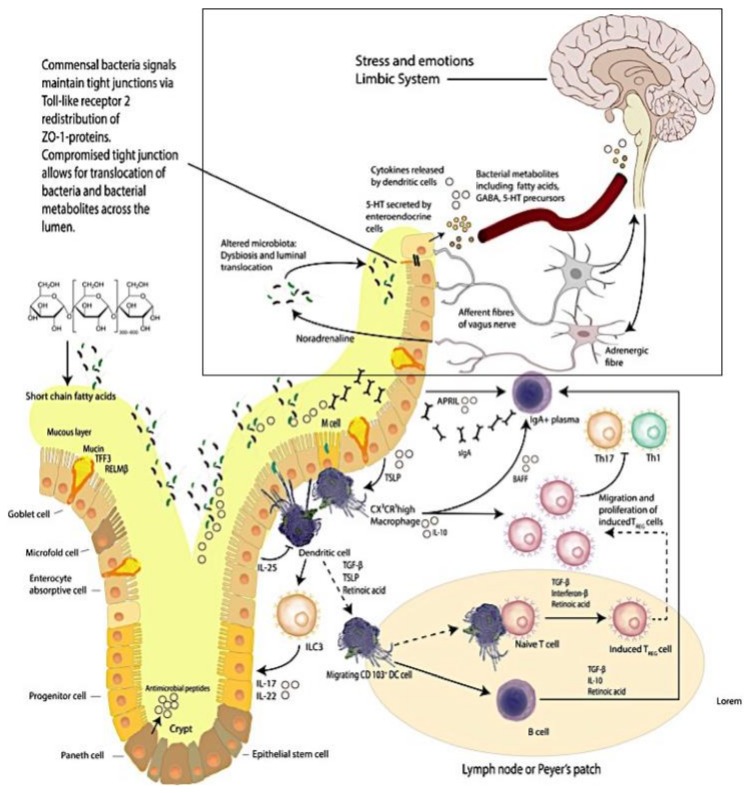
The Brain–Intestinal Microbiome–Intestinal Epithelia–Neuroendocrine-immune–Vagus Nerve–Brain Loop. Mucus production and immune activities delineate the complexity of the intestinal mucosa site. (This figure was adapted from selected reviews [8,30]).

In the gut, intestinal resident macrophages (CX3CR1^hi^) are specialized cells that are involved in antigen presentation to T cells that in turn shape the T cell responses generated [11]. As such intestinal resident macrophages are important participants in contributing and maintaining the steady state equilibrium of mucosal immunity. Macrophages shape mucosal immune equilibrium through the action of phagocytosis eliciting protection from pathobiont translocations across the intestinal epithelium [12] (Figure 1). 

The activated macrophage phenotype is known to present antigens to T lymphocytes, which initiate the controlled and appropriate immune response that is elicited by a recognition signal that responds to microbial proteins [13,14,15] (Figure 1). Studies report that antigen presentation triggers macrophage activities that activate T cells; the activity of macrophages is linked to up regulation of a sequence of cytokines that includes interleukin 1 (IL-1), interferon-alpha (IFN-a) and other cytotoxic proteins [13]. have an important role in maintaining immunological equilibrium [15].

Maintaining immunological equilibrium also involves the phagocytosis of exogenous antigens, cellular debris, insoluble particles and activated clotting factors [15]. It is also noted that intestinal resident macrophage populations sustain mucosal tolerance by contributing to the survival and expanding T lymphocytes already primed toward immunological defense of pathogens [8]. Further, the colitogenic T lymphocyte inflammatory response that occurs in the intestinal mucosa is suppressed by the anti-inflammatory cytokine IL-10 elaborated by intestinal resident macrophages; an activity promoting intestinal mucosa immunological tolerance. The macrophage can be hence envisaged to promote regulatory T cell activities [8]. 

The intestinal epithelia produce soluble protein factors (e.g., thymic stromal lymphopoietin, transforming growth factor-b, and retinoic acid) and also express the integrin ligand semaphorin 7A that undergoes contact-dependent interactions with intestinal macrophages. This activity induces the expression of IL-10 that in turn further promotes intestinal homeostasis. Therefore these cumulative actions combine components of the local innate immune system (i.e., macrophages and dendritic cells and others) and the intestinal epithelia in an interaction that preserves a tolerogenic functional steady state [13]. 

A recent review by Roman and colleagues [16] has progressed the view that macrophages can influence inflammatory disease outcomes and that sustained inflammatory responses can lead to depressive moods. Furthermore it was postulated that antidepressive medications can influence peripheral and brain macrophages skewing them toward anti-inflammatory activities that then can improve cognitive functions [16]. 

### 1.2. The Brain–Intestinal Microbiome/Epithelia–Mucosa–Brain Loop

There are multiple mechanisms in the gut including a mucus layer, antimicrobial peptides and a tight junction protein network that cooperate to continuously preserve local homeostasis and hence ensure that the intestinal epithelial barrier integrity is maintained. Goblet cells secrete mucin to provide a protective coating, provide structural integrity and regulate macrophage and adaptive T cell responses during inflammation [8,17,18,19,20,21,22,23,24,25,26,27,28,29] (Figure 1). 

Mental health is very much intricately linked to physical health [31]. Gastrointestinal dysbiosis is associated with an intestinal epithelial cell barrier dysfunction that can be due to environmental and nutritional triggers, which can be further progressed by the intestinal pathobiont cohort that exacerbates and maintains intestinal barrier in a dysfunctional pro-inflammatory state. It has been recently reported that depression is linked to the exacerbation in gap junction integrity between intestinal epithelial cells (i.e., also termed a leaky gut) is a contentious posit. A recent study has reported that approximately 35 % of depressed individuals exhibited evidence of a leaky gut [32,33]. 

The importance exhibited by the brain–intestine–brain axis is that it provides a bidirectional flow of neuroendocrine-immunological equilibrium control. The intestinal microbiome is thought to exert effects on this axis that significantly impacts the biochemistry of the central and peripheral nervous systems and in turn behavior [34] (Figure 1 (see within the rectangular area)). Studies reporting that depression is accompanied by activation of immune–inflammatory pathways [35] with increased IgM/IgA responses to lipopolysaccharides (LPS) from gram-negative commensal bacteria, indicate that at least in part adverse mood is supported by commensal microbes and or metabolite translocations across the intestinal epithelial barrier [32,33]. We have reported [36] (as have others [37]) that resistant depression can be accompanied by systemic inflammatory states that are posited to originate from intestinal inflammation and the resultant intestinal dysbiosis.

### 1.3. The Vermiform Appendix 

**Figure 2:**
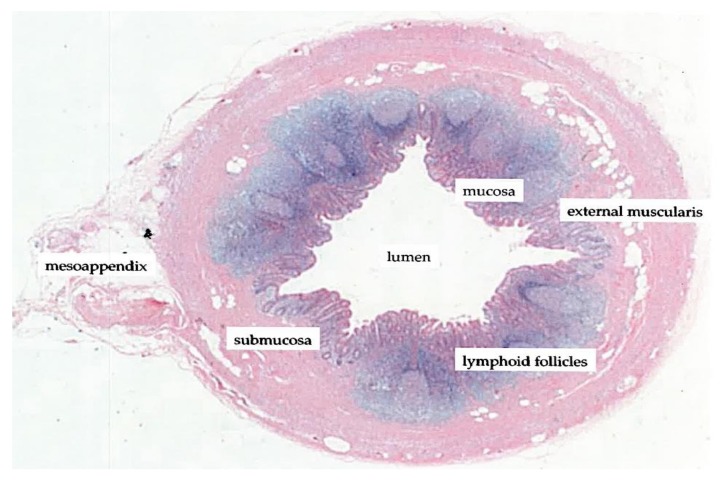
Histological transverse section of the vermiform appendix (adapted/modified from Kooij et al., 2016 [43]).

The vermiform appendix is characterized as a diverticulum of the cecum and delineates the beginning of the colon in the confluence of tanias [38]. The appendix is posterior-medially attached to the cecum, approximately 2 cm below the ileocecal junction. The histological structure of the appendix reflects that of the intestinal wall of the large bowel, in particular with appendiceal structures such as the mucosa, submucosa and lymphoid follicles (Figure 2). 

It has only been recently recognized that the human vermiform appendix is not just a rudimentary part of the intestine, but rather as suggested by numerous studies an organ of immunological importance for the development and preservation of the intestinal immune system [39,40]. Furthermore the importance of the vermiform appendix has been demonstrated to have a direct functional interaction with the intestinal microbiome [41,42].

Reports present the vermiform appendix as an important participant in intestinal ecological microbial contributions and maintenance [41]. This is especially relevant to the ascending colon and the distal small bowel where much of the microbiome is posited to have high metabolic activities. Reports have documented key activities that through co-evolution the human host and the microbial cohort have shaped a tolerant relationship of benefits. These beneficial activities include (i) the intestinal based immune system sustains microbial biofilms in the intestines and is a key constituent of the mutualistic connection between mammals and bacteria [44,45] (ii) biofilms defined as mucilage layers in the gut have been reported to be safe bacterial zones [46], where bacteria and other entities (e.g., fungi) form communal relationships for safeguarding their activities and survival; (iii) the vermiform appendix is very much recognized as an immune tissue [47], with concentrated gut-associated lymphoid tissue; (iv) a recent reported concentrated biofilms contribute to the overall epithelial structure of the vermiform appendix [41,48]; (v) research also shows that biofilms in the intestines are subject to continual turnover an activity that helps to limit bacterial translocations across the intestinal epithelial cells and Peyer’s patches [48], where this mucin turnover activity is rapid (approximately 1–2 h) of any biofilms that adhere to the intestinal epithelia [49].

Early reports through culture dependent studies have demonstrated the presence of several Gram-negative bacilli such as Klebsiella, Enterobacter and *Escherichia coli* whereas Gram-positive cocci were less frequently observed [50]. In a more recent study with patients following an appendectomy showed a comprehensive view of the microbial population within the biofilm of the appendix, this can be determined by high-throughput DNA sequencing [51]. The human appendix was demonstrated to contain members of some fifteen bacterial phyla [51], including Firmicutes (the most dominant), Proteobacteria, Bacteroidetes, Actinobacteria, and Fusobacteria. Moreover, certain oral pathogens not associated with the intestines were also detected in the appendix samples (i.e., Gemella, Parvimonas, and Fusobacterium). This report presents an immunological organ with a significant associated microbial diversity which in part supports the posit that the appendix microbiota has important functions in human health; the biofilm in the appendix acts as a safe house for commensal bacteria, therefore, facilitating re-inoculation of the intestines post a gastrointestinal tract infection as the appendiceal lumen is spared from diarrhoeal clearance [40].

Health interventions that may disrupt the microbiome ecology of the large bowel may lead to disease. A recent study has highlighted that the vermiform appendix may have a significant association with chronic diseases in particular in the development of large bowel cancer following the surgical removal of the appendix [52]. Recently a study of patients clinically treated with antibiotics for appendicitis were reported to have a significant increased risk for large bowel cancer [53]. Moreover, although there is a paucity of studies on the relationship of appendicectomy to mood disorders, an early study has postulated that an appendicectomy may lead to psychological disturbances [54]. An interesting corollary from another study with subjects undergoing an appendicectomy showed that 80% of the participants with inflammatory problems of the appendix were designated as positive for depressive symptoms [55]. Moreover psychological depressive symptoms have been reported to continue post an appendicectomy [56]. These studies overall tend to support the notion that severe disruptions of the intestinal microbiome and the loss of the appendix biofilm may increase the risk of disease including adverse mood dispositions.

### 1.4. Probiotics

Current research continues to draw connections between the intestinal microbiome and environmental factors to sensitivities to the host’s emotional states [57]. In animal models, dysbiosis has been demonstrated to impair vagus signaling which results in reduced protein synthesis in the hippocampus, corrected by rescuing the intestinal microbiome with either specific strains of probiotics [58,59]. Our group has recently demonstrated in a small pilot study [60] with treatment resistant depression (while on SSRI medications) that the administration of a probiotic formulation improved depressive symptoms in a small cohort. An anti-inflammatory response was suggested. A meta-analysis by Ng and colleagues [61] investigating the administration of probiotics to alleviate depressive symptoms was largely inconclusive with an insignificant effect on mood. 

An interesting recent study [62] in a healthy murine model demonstrated that oral administration of probiotic bacteria cell walls stimulated the immune system. This study although preliminary, showed that probiotic bacteria and their cell walls have important immunoregulatory effects on intestinal epithelial cells without an adverse effect on the local metabolic and environment equilibrium. The study by Lemme-Dumit and colleagues [62] showed that cell walls from probiotic bacteria increased immunoglobulin A secreting cells in the intestines and in innate immune cells as well as other tissues such as those of the spleen and peritoneum [62]. This study also demonstrated the capacity that probiotic bacteria provide to stimulate important cellular and immune elements such as the intestinal epithelia and intestinal resident macrophages in the gut [62]. 

## 2. Reprise

We have previously postulated that depressed mood [36] is linked to antagonistic immuno-endocrine control of intestinal homeostasis. The scientific rationale posited suggests that inflammation of the intestines relevant to depressed mood is an effect associated with the intestinal mucosa. Intestinal microbiome adverse shifts that maintain low-level pro-inflammatory activity and intestinal dysbiosis that overwhelm intestinal resident macrophages is posited to play a role in depressed mood. Clinical studies show that the vermiform appendix re-inoculates the proximal colon following excessive pro-inflammatory triggers (e.g., gastrointestinal inflammations, dysentery, antibiotic administration). Reports of a surgically removed chronically inflamed vermiform appendix have been linked to chronic disease developed including depression. 

We postulate that intestinal inflammations may provoke an increased risk for adverse mood disorders in those without a vermiform appendix. In depression the loss of keystone intestinal bacterial species and the incapacity to restore microbial diversity and stability in the proximal colon (i.e., due to an appendectomy) could be an important factor that disrupts the steady state of neuro-endocrine-immunological equilibrium in the intestinal mucosa, especially following the over prescription of antibiotics. We advance the idea that an in situ normal functioning vermiform appendix continually contributes to diversity and stability to the intestinal bacterial cohort over a lifetime. Probiotics that can regulate the functioning of the immune system in the gut may have important plausible implications in improving depressive mood states; dedicated studies are warranted.
